# Simple and fast quantification of DNA damage by real-time PCR, and its application to nuclear and mitochondrial DNA from multiple tissues of aging zebrafish

**DOI:** 10.1186/s13104-017-2593-x

**Published:** 2017-07-11

**Authors:** Shusen Zhu, James A. Coffman

**Affiliations:** MDI Biological Laboratory, Salisbury Cove, ME USA

**Keywords:** DNA damage, Quantitative PCR, Nuclear, Mitochondrial, Aging, Zebrafish

## Abstract

We describe a real-time (rt) PCR-based method of quantifying DNA damage, adapted from the long-run rtPCR method of DNA damage quantification (LORD-Q) developed by Lehle et al. (Nucleic Acids Res 42(6):e41, 2014). We show that semi-long run rtPCR, which generates amplicons half the length of those generated in LORD-Q, provides equivalent sensitivity for detecting low lesion frequencies, and better sensitivity for detecting high frequencies. The smaller amplicon size greatly facilitates PCR optimization and allows greater flexibility in the use of detection dyes, and a modified data analysis method simplifies the calculation of lesion frequency. The method was used to measure DNA damage in the nuclear and mitochondrial genomes of different tissues in zebrafish of different ages. We find that nuclear DNA damage generally increases with age, and that the amount of mitochondrial DNA damage varies substantially between tissues, increasing with age in liver and brain but not in heart or skeletal muscle, the latter having the highest levels of damage irrespective of age.

**Electronic supplementary material:**

The online version of this article (doi:10.1186/s13104-017-2593-x) contains supplementary material, which is available to authorized users.

## Background

The information encoded in the nucleotide sequence of genomic DNA is essential for biological function, and cells employ numerous mechanisms to repair damaged DNA and maintain genomic integrity. Nonetheless, the frequency of genotoxic insults can overcome a cell’s capacity for DNA repair, leading to the accumulation of DNA damage that can in turn lead to cell death or senescence, or transformation to non-functional or pathological cellular phenotypes. The question of how much damage is present in the DNA of a cell, tissue, or organism at different stages of a life cycle or in different environmental or physiological circumstances is central to various fields of biomedicine, including toxicology, oncology, and gerontology; hence, robust, sensitive, and user-friendly methods of quantifying DNA damage are widely called for in biomedical science.

Since lesions that disrupt DNA integrity interfere with DNA replication, DNA damage can be measured by polymerase chain reaction (PCR). Quantitative PCR (qPCR) methods of measuring DNA damage make use of the fact that replication efficiency is proportional to the average frequency of lesions within a defined region, and hence the length of that region [1]. Generally these methods work by comparing the ratios of long and short amplicons obtained from different samples, the latter providing a baseline (virtually damage-free) reference. Originally this approach made use of end-point PCR [2]. More recently a real-time PCR method of using long-run real-time (rt) PCR for DNA damage quantification (LORD-Q) was described which allowed sensitive and robust detection of the number of lesions (including double-stranded breaks, abasic sites, thymine dimers, and 5-hydroxymethyl dC) within a defined sequence [3]. As originally described this method requires amplification of relatively long sequences, ~3–4 kb in length.

Although PCR is straightforward in theory, in practice it often requires extensive optimization to maximize efficiency and specificity, and the ease of optimization decreases with increasing amplicon length. We therefore asked whether the LORD-Q method could be adapted to work with shorter amplicons. We find that semi-long run (SLR) amplicons of ~1.5–2 kb provide sensitivity equivalent to that provided by LORD-Q for detection of low-frequency damage, and better sensitivity for high-frequency damage. Shorter amplicons make PCR conditions less stringent and therefore easier to optimize. To demonstrate the utility of this method, we use it to quantify DNA damage in the nuclear and mitochondrial genomes from multiple tissues of zebrafish of different ages. Unlike most studies in the past that that made such measurements following differential isolation of the mtDNA and nDNA, in our analysis the mtDNA and nDNA were co-isolated, allowing their direct comparison. Our results show that DNA damage varies between tissues, is on average higher in mitochondrial DNA than in nuclear DNA, and generally increases with age.

## Methods

### DNA isolation and purification

Total DNA was purified from zebrafish tissues using E.Z.N.A. tissue DNA kit (OMEGA, USA). DNA quantity and purity were determined by spectrometry using a Nanodrop 1000. The purified DNA (A260/A280 ≥1.8) was stored at 4 °C.

### Primers for the real-time PCR

For comparison of the sensitivity and accuracy of long run (LR) and semi-long run (SLR) PCR of nuclear DNA (nDNA) and mitochondrial DNA (mtDNA), and to examine the difference in the amount of damage in coding region and D-loop regions of mtDNA, we designed 2 pairs of long-run primers for amplicons of 3.5–4 kb, 3 pairs of semi-long-run primers for amplicons of 1.6–2 kb, and 2 pairs of short run primers for amplicons of 55 bp which served as internal undamaged references for normalization (Table 1). All three pairs of primers for nDNA are located in the zebrafish (*Danio rerio*) *aryl hydrocarbon receptor2* (*AHR2*) gene, and the four pairs of primers for zebrafish mtDNA cover different regions. The primers were designed using program NCBI/primer-BLAST (http://www.ncbi.nlm.nih.gov/tools/primer-blast/) and synthesized by Integrated DNA Technologies (IDT, USA) with standard desalting.

**Table 1:** Primers and targets

Primer	Sequence	Amplicon (bp)	Target
AHRLF	GTCCTTGCAGGTTGGCAAATGG	3502	AHR2
AHRLR	GACCTTGTCTGGTTTTCATCCC		
AHRSLF	TCATCCTGTTATCCACCACACTGTTG	1637	AHR2
AHRSLR	TGGTTCTTGGCTACACTGAGATTGAG		
AHRRF	CCAAGGTCCGACATAACTCACTTCTG	55	AHR2
AHRRR	GACATGATGTACTGTGCTGACAACCA		
mtLF	TTAAAGCCCCGAATCCAGGTGAGC	3669	mtDNA coding region
mtLR	TTAGGGGTAGTGAGTTTTGGGTC		
mtSLF	GGATTCCAAGACGCAGCATCACCTG	1978	mtDNA coding region
mtSLR	GGAGCGGCACTTCAAATGGGTCAAG		
mtDLPF	CCTTACACGATTCTTCGCATTCCAC	1939	mtDNA D-loop region
mtDLPR	GGCTTGGCTAGGCGTCTTGG		
mtRF	CGAGGAGCAGGTATCAGGCACA	55	mtDNA coding region
mtRR	GTGGCTTGGCTAGGCGTCTTG		

### Enzymatic digestion of isolated DNA

For quantification of defined lesion frequencies, 5 µg of total DNA isolated from 7 month-old zebrafish brains was subjected to enzymatic digestion with the restriction endonuclease *Pvu*II (for SLR PCR), identified as single cutter for both the targeted nDNA (1637 bp of *AHR2* gene) and mtDNA (1978 bp), or *Nde*I (for LR PCR), identified as single cutter for both the targeted nDNA (3502 bp of AHR2 gene) and mtDNA (3669 bp), using the NEBcutter V2.0 software (http://nc2.neb.com/NEBcutter2). Digestion was carried out at 37 °C for 2 h in a 100-µl volume using 50 units *Pvu*II and *Nde*I (NEB) respectively. For the analysis of DNA-lesion frequency, digested and undigested DNA was diluted and mixed at different ratios (Additional file 1: Table S1) prior to real-time PCR quantification.

### DNA irradiation

1.5 µl of purified DNA from brains of 5-month-old zebrafish was loaded on a piece of parafilm and exposed to ultraviolet C radiation (UVC) at dose of 0.5, 1, 2, 5, 10, 20 and 50 mJ/cm^2^ in a UV crosslinker (UVP HL-2000 HybriLinker, USA). Irradiated DNA was then diluted to 16 ng/µl for nDNA and 2 ng/µl for mtDNA damage assay.

### Real-time PCR

**Fig. 1:**
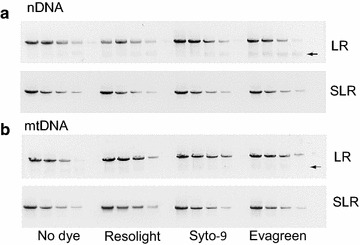
Test of different fluorescent dyes on sensitivity and specificity of LR and SLR PCR. **a** LR and SLR amplicons generated from different quantities of nuclear DNA (nDNA). **b** LR and SLR amplicons generated from different quantities of mitochondrial DNA (mtDNA). *Arrows* indicate non-specific products

Real-time PCR (rtPCR) for DNA damage quantification was carried out in a LightCycler 480 II system (Roche) in 96-well plates. The PCR conditions were optimized for the different primers and different targets to get specific products which were confirmed by melting curve analysis and agarose gels (Fig. 1). The PCR amplification was monitored by real-time measurement of the intercalation of the saturating fluorescent dye into dsDNA. 4 ng of nDNA sample or 1 ng of mtDNA sample was applied in 10 µl of total reaction volume. Standard curves were created by serial dilution of the undamaged control with concentration of 16, 4, 1, 0.25 ng of nDNA and 4, 1, 0.25, 0.0625 ng of mtDNA for quantification of all targets of different sizes. Total DNA from brains of 5-month-old zebrafish (found to have the lowest amount of DNA damage; see Fig. 4) served as undamaged reference. Each sample was assayed in triplicate. For LR and SLR real-time PCR, Platinum Pfx DNA polymerase (ThermoFisher Scientific, USA) was employed and the reactions containing 1X fluorescent dye were performed in 96-well clear plates. Resolight dye was used for all experiments except that which compared the effect of different dyes on the specificity and efficiency of the PCR, which used 1X Resolight (Roche), 1.5 µM SYTO-9 (ThermoFisher Scientific, USA) or 1X EvaGreen (Biotium, USA). For reference quantification (short run), PerfeCTa SYBR Green FastMix (Quanta BioSciences) was used, and the reactions were performed in 96-well white plates. In initial trials comparing Pfx DNA polymerase with hot start Taq polymerase, Pfx was found to be more efficient and more specific than hot start Taq.

### Data analysis

Absolute quantification and second derivative maximum analysis methods were applied for all PCRs (LightCycler 480 Instrument Operator’s Manual, v1.5, 2012). Sample and reference DNA were analyzed side-by-side. DNA damage is quantified by normalizing the concentration values of samples to the concentration values of the internal reference. The resulting values are converted to relative lesion frequencies per 10 kb DNA by application of the Poisson distribution (lesions/amplicon = −ln(A_t_/A_0_)), where A_t_ represents the amplification (normalized concentration) of treated samples and A_0_ is the amplification of untreated or undamaged controls [1].

$$\begin{aligned} Lesions \, per \, 10\;{\text{kb }}DNA\\ &= \left( { - \ln \left( {A_{t} /A_{0} } \right)} \right)\times{\text{10,000}} \, \left[ {\text{bp}} \right]/size \, of \, long \, fragment\;\left[ {\text{bp}} \right] \end{aligned}$$

*long fragment* represents the LR or SLR amplicon.

Statistical analysis of intergroup differences was performed using one-way or two-way ANOVA and Student’s t test.

## Results

### Semi long-run PCR is more efficient than long-run PCR and the products are more specific

We first used standard PCR to compare the specificity of the LR PCR (3–4 kb) used in LORD-Q to that of SLR PCR (1.6–2 kb), as assessed by the appearance of non-specific amplification products on a gel. This was done both without dye added to the reaction, and in the presence of a fluorescent intercalating dye such as is used in real-time PCR. The commonly used fluorescent dye SYBR Green inhibits synthesis of long PCR products, so LORD-Q instead uses the non-inhibitory dye Resolight [3]. We also tested two additional dyes reported to have low inhibitory effects, Syto-9 and Evagreen, which are considerably less expensive than Resolight (340- and 11-fold, respectively). With both nDNA and mtDNA primer sets, SLR PCR yielded a single product in the presence of all three dyes (Fig. 1). In contrast, the LR PCRs generated non-specific products visible as smears or non-specific bands following agarose gel electrophoresis (Fig. 1, arrows), indicating that all three dyes reduce the specificity of LR PCR, with Evagreen having the biggest negative effect. These results indicate that in the presence of intercalating dye SLR PCR is more specific than LR PCR.

### The sensitivity of the SLR real-time PCR based DNA damage assay is comparable to the LORD-Q method

**Fig. 2:**
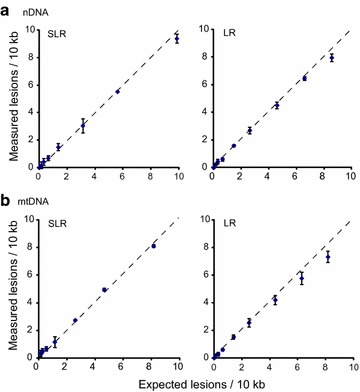
Correlation of calculated and measured DNA lesion frequencies in **a** nDNA and **b** mtDNA. *Error bars* indicate standard deviation of three technical replicates of the PCR

To compare the use of SLR versus LR PCR in a real-time PCR-based DNA damage assay, we digested DNA with restriction endonucleases predicted to make a single cut within the amplified mtDNA and nDNA fragments. The digested DNA was then mixed with undigested DNA at different ratios to generate a standard curve, and both SLR and LR real-time PCR were performed to measure the premade DNA damage. The expected lesion rates were calculated and plotted against the experimentally measured damage (Fig. 2; Additional file 1: Table S1). With both LR and SLR PCR, the measured lesion frequencies tracked the expected frequencies for both the mtDNA and nDNA, albeit more closely with SLR PCR with increasing levels of damage (Fig. 2), suggesting that the latter provides somewhat greater accuracy at higher levels of damage.

**Fig. 3:**
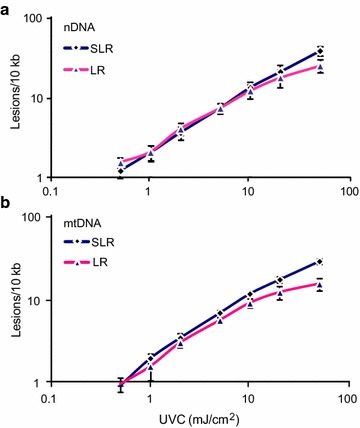
Measurement of DNA damage induced by UVC exposure in **a** nDNA and **b** mtDNA. *Error bars* indicate standard deviation of three experimental replicates, for each of which there were three replicates of the PCR

To further compare the sensitivity of SLR and LR for measuring a range of DNA damage, we subjected naked nDNA and mtDNA (extracted DNA in a small volume of Tris–EDTA buffer) to UVC. DNA damage generated by 5 J/m^2^ (0.5 mJ/cm^2^) UVC should be in the range of 1.5–2 lesions/10 kb [4], and this is what we measured by both methods (Fig. 3; Additional file 1: Table S2). The results also showed that DNA damage increases in a UV dose-dependent manner. At low dose of UVC, the amount of damage in both nDNA and mtDNA detected by SLR PCR is very close to that detected by LR PCR, indicating similar sensitivities. With increasing UVC dose, the signal produced by LR PCR saturated sooner than that produced by SLR PCR (Fig. 3), again indicating that SLR PCR is more sensitive than LR PCR at detecting higher levels of DNA damage.

### Use of SLR rtPCR to measure damage in nuclear and mitochondrial DNA from tissues of zebrafish of different ages

**Fig. 4:**
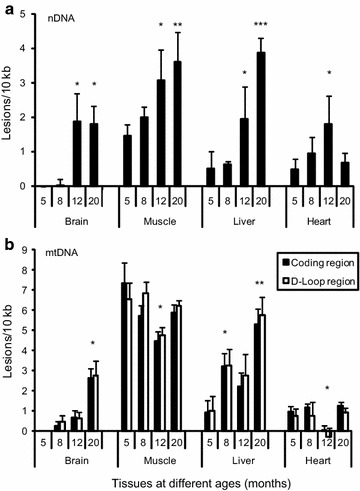
DNA damage measured within **a** nuclear and **b** mitochondrial DNA from different tissues of zebrafish of different ages. *Error bars* indicate standard deviation of three replicate measurements from the same DNA, for each of which there were three replicates of the PCR. Significance (in comparison to 5 months using a two-tailed t test): *p < 0.05; **p < 0.01; ***p < 0.001

As an application we asked whether SLR rtPCR could detect differences in the amount of DNA damage in the nuclear and/or mitochondrial genomes of different tissues of adult zebrafish of different ages, from young adulthood (5 months) through middle age (20 months). Nuclear DNA damage in all the four tissues of zebrafishes was found to increase with age, becoming statistically significant after 12-months (except in 20-month old heart sample) (Fig. 4a). Mitochondrial DNA damage increases with age in brain and liver, with the increase becoming statistically significant at 20-months in brain and after 8-months in liver. No significant change with age was observed in muscle and heart, except in the 12-month old heart sample which had less damage (Fig. 4b). Previous studies have shown that D-loop region of mtDNA, which exhibits a triple-stranded, semi-stable DNA structure during replication, is more vulnerable to oxidative damage and radiation [5, 6]. We therefore asked whether the coding region and the control region containing the D-Loop structure had different amounts of DNA damage. Two-way ANOVA of the results did not show any significant difference in the cumulative in vivo damage displayed by the two regions of mtDNA in brain, muscle and liver, whereas the coding region had slightly higher levels of damage than D-Loop region (p < 0.05) in heart.

## Discussion

Our results indicate that SLR rtPCR provides several advantages to LORD-Q for quantifying DNA damage. With low levels of DNA damage, the sensitivity of SLR rtPCR is comparable to that of the LR rtPCR employed in LORD-Q, whereas with high levels of DNA damage the sensitivity and hence accuracy of SLR rtPCR exceeds that of LORD-Q (Figs. 2, 3). This may be because the LR PCR generates more non-specific product (Fig. 1). In addition, the long amplicons utilized by LORD-Q are inherently less sensitive to high levels of DNA damage than are shorter amplicons: since a single lesion within the amplified region is likely sufficient to block amplification, multiple lesions within a single stretch of DNA are invisible to this method, leading to earlier signal saturation. While shorter amplicons are inherently more sensitive to higher levels of damage, they are less sensitive to low levels. Nevertheless, our results (Figs. 2, 3) demonstrate that SLR provides sensitivity equivalent to LORD-Q for detecting low levels of DNA damage. Since results from previous studies indicate that even shorter amplicons are less sensitive [5], we believe that the ~2 kb employed by our SLR rtPCR method may be optimal for this type of analysis. Importantly, SLR rtPCR makes PCR optimization easier and hence faster, and offers greater flexibility in selection of fluorescent dyes for real time detection (Fig. 1). Finally, whereas the LORD-Q data analysis method requires the use of the Fit Point method to generate the amplification efficiency for the calculation of lesion frequency, our calculation of DNA lesion frequency using normalized concentration values enables the use of the 2nd derivative maximum analysis method (see “Methods”; Additional file 1: Table S3), which offers the advantages of higher accuracy, speed and simplicity.

Applying our method to both the nuclear and mitochondrial genomes of various somatic tissues in aging zebrafish we find that DNA damage accumulates with age, consistent with the widely held view that aging in senescing animals entails loss of genomic stability in somatic tissues. It should be noted that the average levels of DNA damage detected do not reflect per-cell levels of damage, but rather the average level of DNA damage throughout the tissue. Thus the increase in DNA damage seen in several of the tissues probably reflects increasing numbers of senescent or dying cells, rather than increasing loads of DNA damage within healthy cells. Although previous studies have examined the levels of DNA damage and repair capacities in specific tissues at different life cycle stages [7–11], none so far have reported direct comparisons of the amount of DNA damage in different tissues in an adult animal and its change with age. Our measurements showed that both brain and heart have lower nuclear and mitochondrial DNA damage than muscle and liver, consistent with the higher repair capacity that might be expected in the former tissues. The results from muscle and liver are consistent with studies showing that in most tissues levels of DNA damage are higher in mitochondria than in nuclei [11, 12]. Finally, whereas damage in nuclear DNA was found to increase with age in each of the somatic tissues examined, damage in mitochondrial DNA increased in brain and liver, but not in heart or skeletal muscle, wherein the highest levels of DNA damage were found irrespective of age. These results suggest that aging entails increasing levels of nuclear DNA damage in somatic tissues, consistent with what has been found in previous studies [13, 14].

## Additional file

**Additional file 1.** Additional tables.

## Abbreviations

PCR: polymerase chain reaction
rtPCR: real-time PCR
LR: long run
SLR: semi-long run
LORD-Q: long-run DNA damage quantification
nDNA: nuclear DNA
mtDNA: mitochondrial DNA
